# Toll-Like Receptor-4 Disruption Suppresses Adipose Tissue Remodeling and Increases Survival in Cancer Cachexia Syndrome

**DOI:** 10.1038/s41598-018-36626-3

**Published:** 2018-12-21

**Authors:** Felipe Henriques, Magno A. Lopes, Felipe O. Franco, Pamela Knobl, Kaltinaitis B. Santos, Luana L. Bueno, Victor A. Correa, Alexander H. Bedard, Adilson Guilherme, Alexander Birbrair, Sidney B. Peres, Stephen R. Farmer, Miguel L. Batista

**Affiliations:** 1 Integrated Group of Biotechnology, Laboratory of Adipose Tissue Biology, University of Mogi das Cruzes, São Paulo, Brazil; 2 Program in Molecular Medicine, University of Massachusetts Medical School, Worcester, Massachusetts USA; 3 Department of Pathology, Federal University of Minas Gerais, Minas, Gerais Brazil; 4 Department of Physiological Sciences, State University of Maringá, Paraná, Brazil; 5 Department of Biochemistry, Boston University School of Medicine, Boston, Massachusetts USA

## Abstract

Cancer-induced cachexia, characterized by systemic inflammation, body weight loss, adipose tissue (AT) remodeling and muscle wasting, is a malignant metabolic syndrome with undefined etiology. Here, we show that both genetic ablation and pharmacological inhibition of TLR4 were able to attenuate the main clinical markers of cachexia in mice bearing Lewis lung carcinoma (LLC). AT remodelling was not found in LLC tumor-bearing (TB) TLR4^−/−^ mice due to reduced macrophage infiltration and adipocyte atrophy. TLR4^−/−^ mice were also resistant to cold-induced browning of subcutaneous AT (scAT). Importantly, pharmacological inhibition of TLR4 (Atorvastatin) reproduced the main protective effect against AT remodeling found in TLR4^−/−^ TB mice. Moreover, the treatment was effective in prolonging survival and attenuating tumor mass growth when compared to non-treated-TB animals. Furthermore, tumor-induced elevation of circulating pro-inflammatory cytokines was similarly abolished in both genetic ablation and pharmacological inhibition of TLR4. These data suggest that TLR4 is a critical mediator and a promising target for novel anti-cachexia therapies.

## Introduction

Cancer cachexia syndrome is characterized by systemic inflammation, body weight loss, remodeling of adipose tissue (AT), and skeletal muscle wasting^1^. Consequently, it results in reduced quality of life, decreased survival, and increased complications due to cancer treatment. Cachexia is the main cause of death in approximately 20% to 30% of all patients with cancer^2^. In cancer cachexia patients, impairment of AT lipid metabolism has been demonstrated and longitudinal studies have established that AT loss precedes muscular atrophy^3^. The changes that characterize AT remodeling comprise: an increase in infiltrated inflammatory cells^4,5^, extracellular matrix rearrangement^4,6^ and the appearance of beige cells^7^ in subcutaneous AT (scAT). The physiological role and consequences of cachexia-induced browning are not known. The current consensus is that multiple factors contribute to cancer cachexia, and therapy requires combinational strategies^8^. Interestingly, pharmacological intervention aimed at attenuating the AT remodeling in cachexia have shown satisfactory results, particularly in mitigating tumor growth and increasing animal survival^9^.

A plethora of evidence from both patients and animal studies suggest a compelling link between activation of the inflammatory pathways and development of cancer cachexia^10,11^. In fact, systemic inflammation has been proposed as a critical feature of cancer cachexia, and anti-inflammatory strategies are considered central to the therapy^10^. It is likely that pathways responding to similar pro-inflammatory cytokines that mediate both sterile and infectious inflammation are critical in cancer cachexia. The toll-like receptor (TLR) system is potentially one such pathway. TLRs are required for pathogen recognition by the innate immune system^12^. Among TLRs, TLR4 acts as a receptor for lipopolysaccharides (LPS), and associates with the myeloid differentiation protein 2 (MD2) to form a complex to interact with LPS^13^. The stimulation of TLR4 by LPS induces the release of critical proinflammatory cytokines that are necessary to activate potent immune responses^12,13^.

In a cancer cachexia animal model, genetic ablation of TLR4 elicited a less severe cachexia with an accompanying lower body weight loss, greater lean body and fat mass, and clinical evidence of reduced wasting compared with the age and weight-matched WT mice^11^. More recently, an elegant study showed that TLR4 is a crucial mediator of cancer-induced muscle wasting due to its integration of catabolic signaling by directly activating muscle protein degradation and indirectly increasing cytokine release^10^. Thus, TLR4 may be a critical therapeutic target for cancer cachexia. The role of the TLR4 pathway in AT remodeling during cancer cachexia development remains unexplored.

Based on these considerations, the present study was designed to further investigate the role of TLR4 on AT remodeling and cachexia development. Using a genetic ablation and pharmacological inhibition of TLR4 we demonstrate that the TLR4 pathway plays an essential role in modulating both thermogenic and pro-inflammatory pathways in fat. Suppression of TLR4 signaling results in a robust resistance to AT remodeling, reduced cachexia, and increased survival. Our study sheds light on TLR4 pathway as a promising target for therapeutic intervention for cachexia.

## Results

### TLR4 deletion attenuates AT remodeling induced by cancer cachexia in TB-mice

**Figure 1:**
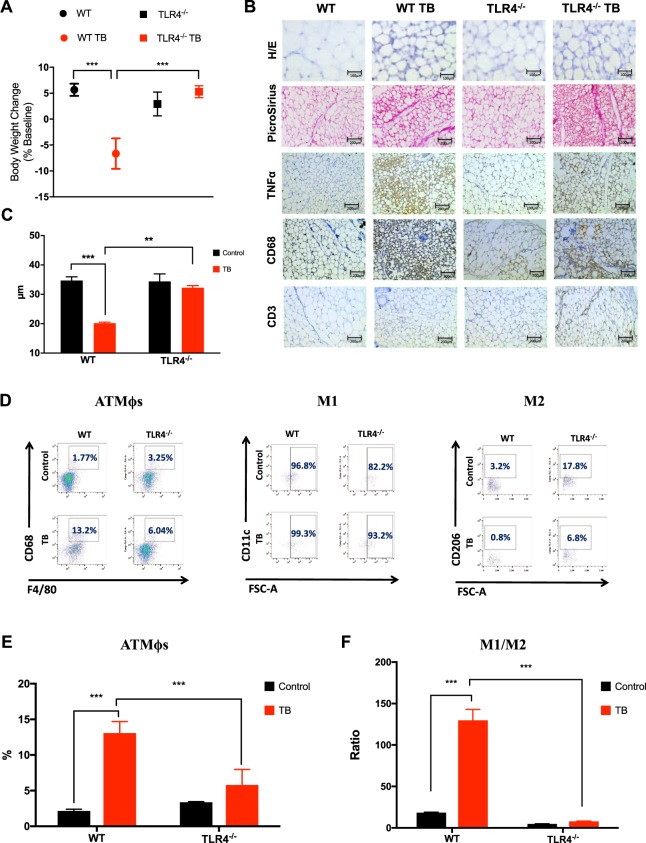
TLR4 deletion attenuates scAT remodeling during cancer cachexia syndrome. (**A**) Wild-type C57BL/6 and TLR4^−/−^ mice (8-week old male) were inoculated with LLC tumor cells. Body weight change (excluding tumor weight) was evaluated after 28 days before inoculated tumor cells. N = 10 per group. (**B**) Histologic sections of scAT in different experimental groups. Histological staining for H/E and picrosirius red were performed, and also, immunohistochemistry for inflammatory profile (TNFα) and immune cell markers (CD68 and CD3). N = 5 per group. (**C**) The size of adipocytes (cell diameter) from WT and TLR4^−/−^ mice was quantitatively analyzed (500 adipocytes were measured for each group) after the experimental protocol. N = 5 per group. (**D**) Stromal vascular fractions (SVF) were isolated from scAT by collagenase digestion for each different group. Flow cytometric analysis of SVF was conducted using fluorescent-conjugated antibodies against CD68, F4/80, CD11c, CD206. Adipose tissue macrophages (ATMϕs) were defined as CD68^+^F4/80^+^ subpopulations and displayed the values as percentage of your respective groups. M1 and M2 ATMϕs were defined as CD68^+^F4/80^+^CD11c^+^CD206^−^ and CD68^+^F4/80^+^CD11c^−^CD206^+^, respectively. Representative flow cytometric dot plots showing the percentage of ATMϕs. N = 4 per group. (**E**) Quantification of double-positive cells for CD68^+^F4/80^+^ related to dot plots showed in (**D**). (**F**) M1/M2 ATMϕs ratio in the scAT. Scale bars, 100 μm and 200 μm. Graphs show the mean ± SEM. Statistical significance was determined by two-way ANOVA. **P < 0.01; ***P < 0.001.

During the development of cancer cachexia, AT remodeling arises from morphofunctional and inflammatory changes that result in AT dysfunction. Consistent with these observations, we used the Lewis Lung Carcinoma (LLC) cell line to induce cancer-associated cachexia *in vivo*^10,11^. Additionally, we also determined whether LLC tumor induces AT remodeling through activation of TLR4-mediated AT immunometabolism changes. Thus, we utilized the existing TLR4^−/−^ mice^14^ to examine the role of TLR4 in cancer cachexia. 28 days after LLC cell inoculation a 12% reduction in body weight was measured in tumor-bearing wild-type (WT TB) mice, a classic sign of cachexia (Fig. 1A). However, in the TLR4^−/−^ TB mice there was no weight loss, without any differences in tumor growth (Fig. S1). No pulmonary metastasis was detected in any of the evaluated TB groups.

Adipocyte atrophy in scAT, demonstrated by a marked reduction in adipocyte size (58.2%), and significantly increased fibrosis, demonstrated by total collagen content in the extracellular matrix (Fig. 1B,C), were also observed in WT TB mice. Furthermore, the presence of CD68 and TNF-α positive staining in scAT was found, while no significant difference in CD3 positive cells in scAT was observed (Fig. 1B). On the other hand, tumor-bearing TLR4^−/−^ TB mice presented less adipocyte atrophy and a reduction in the number of TNF-α and CD-68 positive cells in scAT when compared to WT TB group (Fig. 1A–C). Taken together, the effects of cachexia on inducing AT remodeling were attenuated in TLR4^−/−^ TB mice.

To better understand AT inflammation and the role of macrophage polarization during cancer-associated cachexia, we evaluated AT macrophage (ATMϕs) profiles in scAT from WT and TLR4^−/−^ mice during development of the cachexia syndrome. In this case, scAT demonstrated an increase in ATMϕs (6.0-fold) (Fig. 1D,E) with an enhancement in M1 (7.0-fold) and a decrease in M2 ATMϕs in the WT TB mice (Fig. 1D). In contrast, the TLR4^−/−^ TB mice presented a consistent attenuation in ATMϕs infiltration (55.3%) and a decrease in the ratio of M1/M2 macrophages (93.2%) in the scAT when compared to WT TB mice (Fig. 1D,E). In models of LLC-induced cachexia, ATMϕs polarization tends to be directed towards an M1 phenotype, making these results even more significant. In addition to the macrophage polarization, the proportion of M2 macrophage was higher in the TLR4^−/−^ (4.5-fold) and TLR4^−/−^ TB (8-fold) mice when we compared with their respective controls (Fig. 1D). Therefore, the attenuation of TLR4 was associated with a reduction in the recruitment process of ATMϕs, thus playing an interesting role in the development of cancer cachexia syndrome.

### Triglyceride turnover is not affected during cachexia in TLR4^−/−^ TB-mice

**Figure 2:**
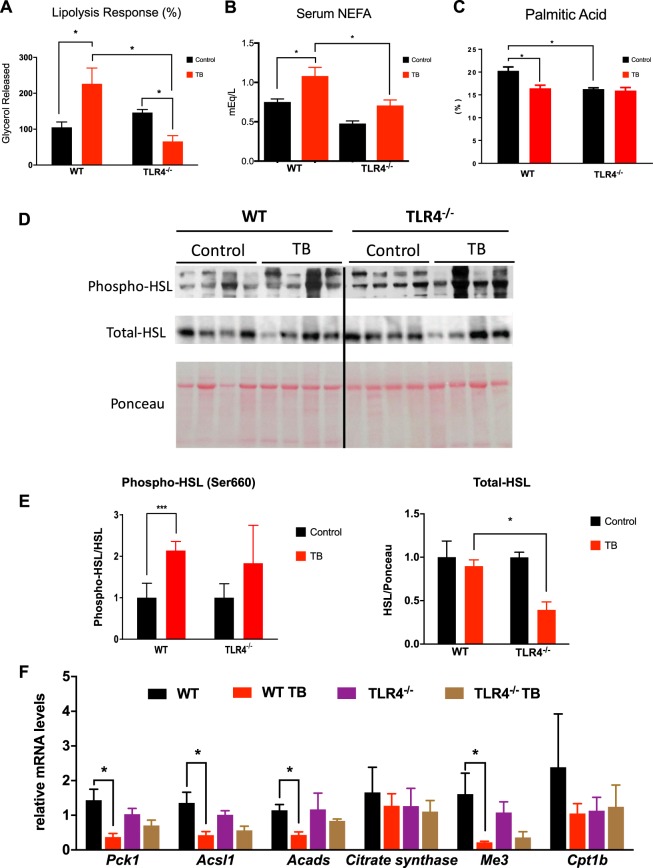
Reduced triglyceride (TG) turnover is observed in TLR4^−/−^ TB-mice. (**A**) Primary adipocytes from scAT were incubated in the presence (stimulated) or absence (basal) of isoproterenol (10^−5^M) for 1 hour. Graph shows lipolysis response assessed by the ratio of glycerol released (nmol/10^4^ cells) relative to the basal condition. N = 4 per group. (**B**) Serum concentrations of non-esterified fatty acids (NEFA) and (**C**) % of palmitic acid in scAT were performed for each different group. N = 4 per group. (**D**) Immunoblot analysis for components of lipolysis. scAT lysates were immunoblotted for phospho-HSL (Ser660) and total HSL (**E**) Densitometric evaluation of phospho-HSL (Ser660) and total HSL. Ponceau staining was analyzed as a loading control. N = 4 per group. (**F**) qRT-PCR was performed to quantitate *Pck1*, *Acsl1*, *Acads*, *Citrate Synthase*, *Me3* and *Cpt1b* mRNA levels in scAT from the different groups. N = 5 per group. Graph show the mean ± SEM. Statistical significance was determined by two-way ANOVA. *P < 0.01; ***P < 0.001.

Considering the attenuation of the AT remodeling seen in the TLR4^−/−^ TB mice in response to cachexia, the next step was to evaluate the cachectic response of primary adipocytes (scAT) after isoproterenol (ISO)-stimulus, to evaluate the lipolytic response. During the development of cachexia, an increase in glycerol release (1.2- fold) was observed in the WT TB group compared to the WT group (Fig. 2A). On the contrary, adipocytes in the TLR4^−/−^ TB mice showed lower lipolytic response when compared to the WT TB group (Fig. 2A).

Once the attenuation of cachexia-induced lipolysis was shown in the TLR4^−/−^ TB mice, serum concentrations of non-esterified fatty acids (NEFA) and palmitic acid levels from the scAT in the different experimental groups were evaluated (Fig. 2B,C). A significant increase was found in NEFA serum concentrations after the cachexia induction in WT TB mice. However, the serum concentration of NEFA was reduced by 36.3% in the TLR4^−/−^ TB mice (Fig. 2B) when compared to the WT TB mice. Palmitic acid levels in scAT were reduced by 18.9% in the WT TB mice when compared with the WT mice (Fig. 2C). Interestingly, the TLR4^−/−^ group had lower levels of palmitic acid in the scAT when compared to the WT group and no difference was observed between the TLR4^−/−^ and TLR4^−/−^ TB mice (Fig. 2C).

The main lipolytic enzymes were also evaluated. Hormone-sensitive lipase (HSL) showed an increased phosphorylation at ser-660 in the WT TB group, without any alteration in the TLR4^−/−^ TB group (Fig. 2D,E). In this scenario, since triglyceride (TG) turnover is affected by cachexia and partially diminished by TLR4 deletion, we evaluated genes involved in fatty acid metabolism in adipocytes. In general, the effect of cachexia on the expression of scAT metabolic markers was reduced in the absence of TLR4. In particular, cachexia reduced expression of *Pck1*, *Acsl1*, *Acads* and *Me3* in the WT TB, but not in the TLR^−/−^ TB mice (Fig. 2F).

### TLR4 deletion reduces AT browning and p38MAPK signaling in TB mice

**Figure 3:**
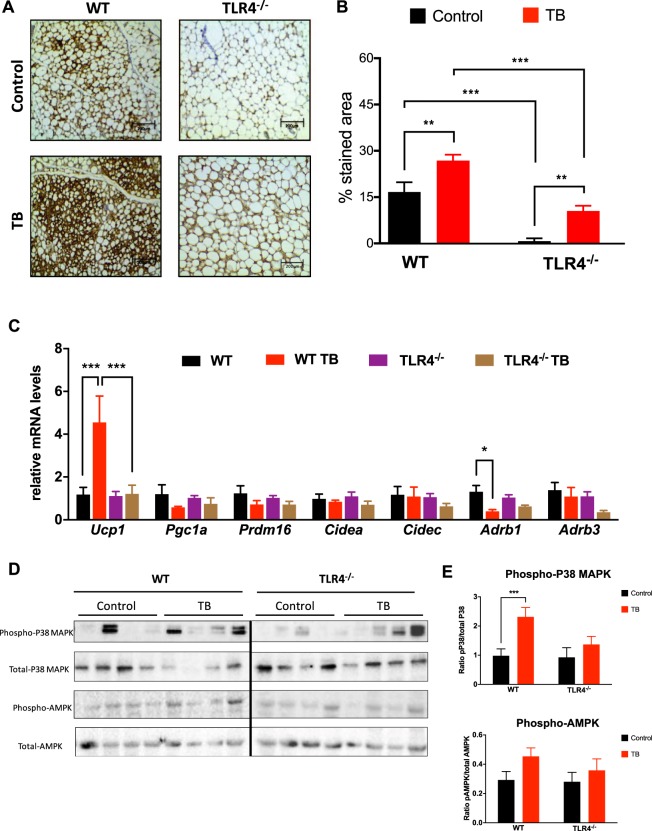
TLR4 deletion reduces browning effect in TB mice throughout p38MAPK pathway. **(A**) Representative images of UCP1 staining of scAT from the different experimental groups. N = 5 per group. (**B**) Total quantification of UCP1 staining. (**C**) qRT-PCR was performed to quantitate *Ucp1*, *Pgc1a*, *Prdm16*, *Cidea*, *Cidec*, *Adrb1* and *Adrb3* mRNA levels in scAT from the different groups. N = 5 per group. (**D**) Depicted are representative immunoblots to detect phospho-p38MAPK, p38MAPK, phospho-AMPK and AMPK levels in scAT from the different experimental groups. N = 4 per group. (**E**) Densitometric evaluation of protein levels (phospho/total). Scale bars, 200 μm. Graphs show the mean ± SEM. Statistical significance was determined by two-way ANOVA. *P < 0.05; **P < 0.01; ***P < 0.001.

To assess whether TLR4 mediates AT energy expenditure, thermogenic markers were measured in scAT. We found that UCP1 staining was increased (1.3-fold) in TB mice (Fig. 3A,B). Surprisingly, TLR4^−/−^ and TLR4^−/−^ TB mice showed a significant reduction in UCP1 staining (Fig. 3A,B). Corroborating this data, the gene profile of the browning “signature” from scAT showed a marked upregulation of *Ucp1* levels in the WT TB mice, but in the TLR4^−/−^ TB mice there was a significant attenuation in the browning profile, as illustrated in Fig. 3C.

Adaptive thermogenesis and UCP1 expression are mainly regulated by sympathetic tone through β-adrenergic signaling and cAMP levels, which can be directly sensed by protein kinase A (PKA) and thus lead to direct or indirect activation of p38 MAPK. Although cachexia induced phosphorylation of p38 MAPK in the WT TB group, there was no change in the TLR4^−/−^ TB group. (Fig. 3D,E). There were also no changes in phosphorylation of AMPK (Fig. 3D,E) and PKA substrates in any of the experimental conditions evaluated (Fig. S2) in the TLR4^−/−^ TB mice.

### TLR4 is required for cold-induced browning of inguinal white adipose tissue

**Figure 4:**
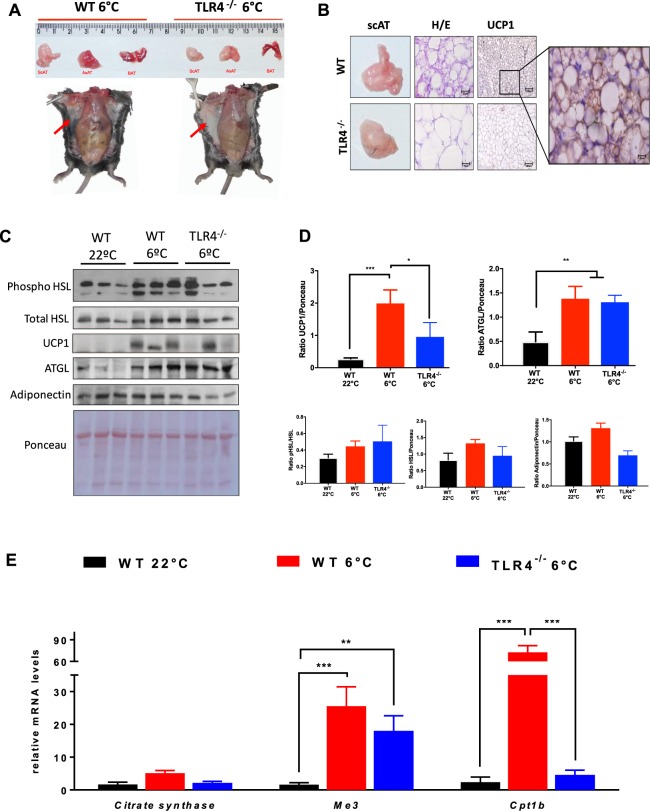
TLR4 is required for cold-induced browning phenotype. (**A**) Representative image of WT and TLR4^−/−^ mice were single-caged and housed at room temperature (23 °C) and cold exposure (6 °C) for six days (N = 3 per genotype and per condition). (**B**) Representative image of UCP1 staining in scAT across the different genotypes. (**C**) Depicted are representative immunoblots to detect phospho-HSL, total HSL, UCP1, ATGL and Adiponectin. N = 3 per group. (**D**) Densitometric evaluation of phospho-HSL, total HSL, UCP1, ATGL and Adiponectin levels. (**E**) qRT-PCR was performed to quantitate *Citrate synthase*, *Me3* and *Cpt1b* mRNA in scAT from the different groups. N = 3 per group. Scale Bars, 25 μm and 100 μm. Graph shows the mean ± SEM. Statistical significance was determined by one-way ANOVA. *P < 0.05; **P < 0.01. ***P < 0.001.

Having shown that TLR4 is required for full browning of scAT during cancer-associated cachexia, we subjected WT and TLR4^−/−^ mice to chronic cold (6 °C) challenge to see if TLR4 is required for general adaptive thermogenesis. There was a marked reduction in cold-induced browning in the TLR4^−/−^ mice compared to the WT mice (Fig. 4A,B). Interestingly, expression of proteins linked to the lipolytic pathway (Phospho HSL and ATGL) were the same across both groups (WT 6 °C and TLR4^−/−^ 6 °C), when compared to WT at 22 °C (Fig. 4C,D). Therefore, we did not see any UCP1 protein enhancement in the TLR4^−/−^ mice after cold exposure. We observed a statistically significance decrease in UCP1 expression in the TLR4^−/−^ 6 °C group when compared to WT 6 °C group (Fig. 4C,D). Although oxidative metabolism (TCA cycle) was stimulated by chronic cold exposure in both groups (WT and TLR4^−/−^), an increase in *Cpt1b* gene expression was evidenced only in the WT group, with no change in the TLR4^−/−^ mice (Fig. 4E).

### Atorvastatin treatment increases survival and attenuates browning

In order to propose a translational approach and to correlate the data with the genetic model, WT TB mice were treated with a 3-hydroxy-3-methyl-glutaryl-CoA (HMG-CoA) reductase enzyme inhibitor, atorvastatin (ATOR). In recent years^15–17^, the anti-inflammatory and pleiotropic effects of this drug have been described, such as inhibition of NFκB activation and downregulation of *Tlr4* and *Myd88* gene expression. We also confirmed the downregulation of *Tlr4* after ATOR treatment in scAT, as illustrated in Fig. S3.

**Figure 5:**
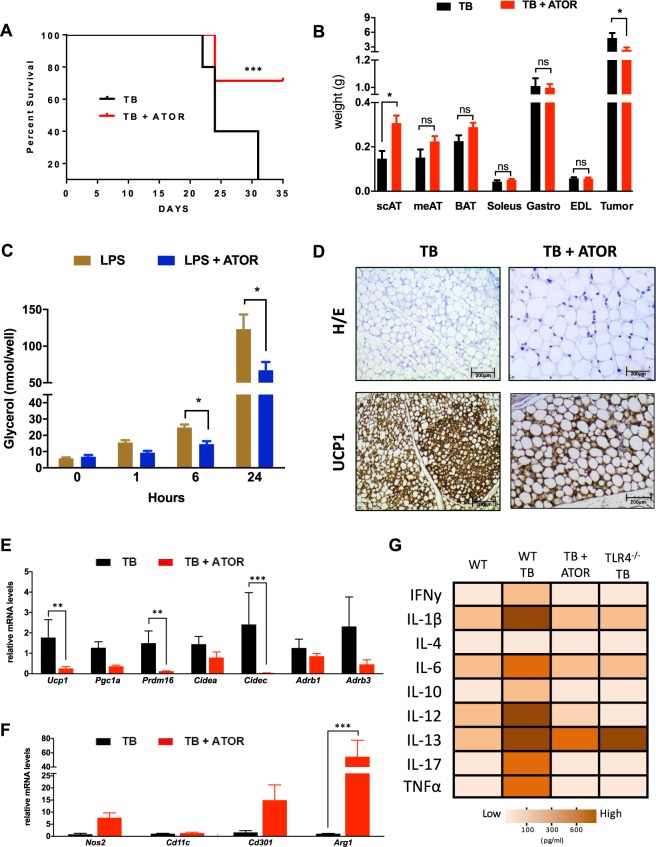
Atorvastatin treatment increases survival and improved cachexia-remodeling in scAT. (**A**) Kaplan-Meier survival curves show a statistically significant difference (P < 0.05) in survival between the Tumor Bearing (TB) mice (N = 15 per group) and Tumor Bearing + ATOR treatment (TB + ATOR) mice (N = 15 per group). (**B**) Adipose tissue, muscle and tumor weights at study end. (**C**) Time course of lipolysis in 3T3-L1 induced by LPS (100 ng) and LPS + ATOR treatment (100 μM). (**D**) Immunohistochemical analysis for UCP1 in scAT. (**E**) qRT-PCR was performed for *Ucp1*, *Pgc1a*, *Prdm16*, *Cidea*, *Cidec*, *Adrb1* and  *Adrb3* (**F**) *Nos2*, *Cd11c*, *Cd301* and *Arg1* (M1 and M2 macrophage polarization markers) for mRNA quantification in scAT from the different groups. N = 5 per group. (**G**) Heat map representing the circulating pro-inflammatory cytokines in the serum from the different experimental groups. White to brown scale depicts cytokine levels in pg/ml. Data were analyzed using the Bio-Plex manager software. N = 8 per group. Scale bars, 200 μm. Graph show the mean ± SEM. Significant differences were determined using Student’s *t*-test (**B**,**E**,**F**) and two-way ANOVA (**C**). Subcutaneous AT (scAT); Mesenteric AT (meAT); Brown AT (BAT); Extensor Digitorum Longus Muscle (EDL). *P < 0.05; **P < 0.01; ***P < 0.001.

To evaluate if pharmacological attenuation of TLR4 would have an impact on the clinical features of cachexia, WT TB mice were treated with ATOR (Fig. 5). The treatment was effective in prolonging the mice survival (Fig. 5A) and also in attenuating scAT atrophy (2.0-fold) and tumor mass growth (49.7%) when compared to a TB untreated group (Fig. 5B). Other markers of the cachexia syndrome evolution in the animal model were attenuated in those treated with ATOR. Body weight (BW) plateau, which represents the highest value of body weight observed in the experimental period, showed an increase of 32% in the treated animal when compared to the WT TB group. The tumor of ATOR-treated animals appeared later than the tumor of the non-treated animals (Table S1). Lipolysis was also evaluated in 3T3-L1 cells. Cells that were stimulated with lipopolysaccharide (LPS) and treated with ATOR showed a reduced lipolytic response to the LPS stimulation when compared with untreated cells, showing a significant reduction at 6 hours (41.3%) and 24 hours (45.6%) as illustrated in Fig. 5C.

Interestingly, ATOR was also able to reduce cachexia-induced browning and atrophy of scAT (Fig. 5D,E). Furthermore, the profile of the signature browning genes from scAT showed marked downregulation of *Ucp1* (85.5%), *Prdm16* (91.7%) and *Cidec* (98.3%) after ATOR treatment in TB mice, as shown in Fig. 5E. In respect of ATMϕs polarization, we analyzed the classical markers for M1 (Nos2 and CD11c) and M2 (Arg1 and CD301) in the scAT. No significant difference was observed in mRNA for M1 markers (despite a strong trend). However, Arg1 expression increased 5.8-fold in the TB + ATOR mice. This suggests that ATOR treatment appeared to reverse the previously described M1 ATMϕs polarization, a similar effect to that which we found in the TLR4^−/−^ mice (Fig. 5F).

During cancer cachexia syndrome, systemic inflammation plays a key role in the scAT remodeling, directly influencing the composition of the immune system present in AT, and, consequently, the patient’s immune response. In the present study, we demonstrated that WT TB mice present high plasma concentrations of proinflammatory cytokines in response to cancer-associated cachexia (Fig. 5G). However, after pharmacological treatment with ATOR, it was possible to see a significant decrease in almost all these cytokines, returning the plasma concentration levels to a degree comparable with the WT group (Control group - without cachexia). These results demonstrate that treatment with ATOR improved the inflammatory profile of the tumor-bearing mice and ameliorated some general cachexia parameters after ATOR treatment similarly to the values found in the TLR4^−/−^ TB mice (Table S1).

## Discussion

Cancer-associated cachexia is a complex metabolic state accompanied by poor quality of life, high mortality and also resistance to chemotherapy^18–20^. Several clinical interventions have sought to improve cachexia through anti-inflammatory drugs and insulin sensitizers but studies have shown limited results^21,22^. Circulating factors produced by the tumor and/or host itself may play an important role in therapeutic treatment for cachexia. Once the factors are identified, they can be targeted for therapeutic intervention.

During the development of cancer cachexia, AT remodeling occurs predominantly through morphofunctional rearrangement that is an endpoint process associated with immune-metabolic dysfunction of such tissue^4,5,9,18,23^. In WT TB mice, body weight loss and AT mass wasting were detected, as well as a set of features related to AT remodeling. Interestingly, the TLR4^−/−^ TB animals were shown to be protected against the effects of cachexia, particularly in relation to an absence of weight loss and AT wasting. In obesity (in humans and in mice models)^24–26^ a close link between inflammation, over-production of extracellular matrix (ECM) components, the development of fibrosis and consequent tissue remodeling has been demonstrated in the liver and kidneys. In these organs, there is mounting evidence that TLR4 acts as a key regulator in fibrogenesis^27–29^, a condition that may or may not be related to TLR4^−/−^ ATMϕs response. However, the possible involvement of TLR4 and ECM remodeling in cancer cachexia was not a focus of this study and needs further analysis.

Another important aspect of cancer cachexia-induced AT remodeling is the establishment of AT inflammation that was recently shown to be characterized by increased recruitment of ATMϕs, including activated M1 and M2 macrophages^5^. In fact, ATMϕ infiltration of scAT has consistently been demonstrated in different experimental models^30^. This scenario has also been demonstrated in patients with cancer and cachexia^4,5^. However, the polarization of ATMϕs in scAT has only recently been addressed^5,7^. In this regard, we extended these findings by presenting in greater detail that in cachexia induced by LLC cells, ATMϕs polarization tends to be directed to an M1 phenotype. On the other hand, the TLR4^−/−^ TB mice presented a consistent attenuation in ATMϕs infiltration in scAT, but in this phenotype, the polarization tended towards an M2 phenotype. The exact mechanism by which TLR4 deficiency affects AT inflammation remains to be explored and would require the generation of an adipose tissue-specific knockout mouse model for TLR4.

Regarding the morphological and inflammatory dysfunction that results in AT remodeling in response to cachexia, TG turnover is the most well-characterized metabolic disorder associated with this condition^4,5,31^, with increased lipolysis being the mechanism best described in the literature^23,32^. Higher adipocyte lipolysis was observed in the cachectic WT mice, which is in line with recent studies demonstrating that AT lipolysis represents a key factor involved in weight loss in cancer cachexia in both animals^23,32^ and human patients^33^. However, adipocytes from the TLR4^−/−^ mice were less affected by cachexia and showed a very attenuated response to ISO-stimulation.

As TLR4^−/−^ TB mice were found to be resistant to the lipolytic effects induced by cachexia, we also evaluated some parameters related to the cycle of NEFA release from the breakdown of stored TG and re-esterified to TG (TG turnover). In human patients with cachexia, enhanced TG turnover activity in AT has been shown, as determined by a metabolic labeling assay^20^. In our study, WT TB animals showed a reduction of palmitic acid level in the scAT, corroborating the other results regarding the increase in fatty acids associated with cachexia. Interestingly, this process showed less intensity in TLR4^−/−^ TB mice, including in respect of AT morphofunctional preservation. These data substantiate those found in some recent studies that demonstrated an impairment of TG turnover by cachexia^5,32^. However, it should be noted that the TLR4^−/−^ animals already had lower levels of palmitic acid in the scAT compared to the WT animals. Thus, whether or not this fact is associated with metabolic repercussions requires further investigation.

In general, the effect of cachexia on expression of AT metabolic markers was attenuated in the absence of TLR4, particularly in genes related to glyceroneogenesis and TG reesterification. A study by Pang *et al*. proposed that TLR4 might play a role as a physiological regulator of fuel metabolism^34^. In their study, TLR4^−/−^ mice showed lipid abnormalities during fasting that might result from increased TG mobilization, possibly through increased fatty acid reesterification in scAT, decreased fatty acid oxidation, and/or increased *de novo* lipogenesis in key metabolic tissues. These results corroborate those presented in this study, demonstrating that, in addition to reduced lipolysis, TLR4 deletion may also be involved the avoidance of the deleterious modifications in fatty acid reesterification, induced by cachexia.

It has recently been shown that cachexia induces AT browning in addition to changes in immune-modulatory activity. In this scenario, chronic inflammation and β-adrenergic activation of thermogenesis functionally cooperate in the pathogenesis of cachexia^3,7^. In the present study, LLC-induced cachexia resulted in a consistent browning phenotype (morphology) of scAT followed by a marked upregulation of *Ucp1* levels. These results are in line with those presented in other studies with the same experimental model^35^.

Interestingly, TLR4^−/−^ TB mice showed a significant reduction in AT browning, as well as in the levels of phosphorylation of p38 MAPK, both induced by cachexia. The browning phenotype induced by cancer cachexia might, therefore, be activated indirectly by p38 MAPK, and the presence of TLR4 appears to be important in this process. Recently, it was demonstrated that phosphorylation of p38 MAPK activity was higher in the UCP1 positive axillary AT of cachectic (K5-SOS) animals^7^. Adaptive thermogenesis and UCP1 expression are mainly regulated by sympathetic tone through β-adrenergic signaling and cAMP levels, which can be directly sensed by protein kinase A (PKA) and thus lead to direct or indirect activation of p38 MAPK^36,37^. However, a well-designed recent study using experimental models demonstrated that full activation of adipose browning, including *Ucp1* expression, in mouse and human cachexia seems to be variable, and the functional contribution to overall energy coats needs to be determined^23^.

The reduction of browning induced by cachexia has been demonstrated in different studies, through treatment with cyclooxygenase-2 inhibitor (Sulindac), β3-adrenergic receptor antagonist (SR59230A)^7^ and a knockout model for parathyroid hormone-related protein (PTHrP)^38^. However, none of these studies have shown results on survival, which is a key parameter to verify the pre-clinical efficacy of pharmacological intervention.

Taking into account the beneficial effects found in the genetic model (TLR4^−/−^ TB), and to take a translational (preclinical) approach that would include evidence on survival, we chose to treat the animals with an already well-established drug with a well-characterized TLR4 antagonist effect (ATOR). Some elegant studies have consistently shown that ATOR has an inhibitory effect on the TLR4 pathway^15,39–43^. Another important criterion for drug choice was its direct effect on the down-regulation of this receptor^39–41^ as well as regulation of downstream pathways^15^ and target cytokines^42,43^. Additionally, the fact that it is already well-established in the market, in addition to presenting a very favorable toxicity profile, was also considered^44,45^.

We demonstrated that ATOR treatment was effective in increasing the survival of the cachectic mice, in addition to attenuating the main signs of cachexia syndrome, an effect also observed in the genetic TLR4^−/−^ model. In cachexia, systemic inflammation is highly dependent on the patient’s immune response^46^ and, at least in experimental models, IL-6, TNF-α, and IL-1β are major contributors to the wasting syndrome^6,47^. The major inflammatory cytokines were abrogated in the same way, both in TLR4^−/−^ and pharmacological inhibition of TLR4 in TB mice. In fact, systemic activation of TLR4 increases cytokine synthesis and release from various host cells as an innate immune response^10^. Moreover, a recent study showed the presence of elevated levels of TNF-α and IL-6 in animals bearing-LLC tumor, which was attenuated in TLR4^−/−^ mice in the same experimental condition (cachexia)^10^, corroborating the findings of our study.

Moreover, in addition to the anti-inflammatory and protective effects of skeletal muscle mass previously demonstrated^10^, we have shown that TLR4 activation is “directly” involved in metabolic disorders in AT such as increased TG turnover and browning phenotype, both relevant parameters involved in the remodeling and dysfunction of AT induced by cachexia. We showed in the present study that TLR4 disruption attenuated AT remodeling and metabolic dysfunction during the syndrome, thus suggesting a possible therapeutic target for cancer-induced cachexia.

Some limitations of this study should be acknowledged. First, most of our findings are based on the data obtained from TLR4 whole-body knockout mice. Global TLR4 targeting extends our knowledge of the role of this receptor in AT remodeling caused by cancer cachexia. The TLR4 null mouse model has a significant advantage compared with other knockouts since it has prolonged survival from cachexia. The global knockout, however, did not allow us to track TLR4 function at specific developmental stages of cachexia. Second, although some well-designed studies have also demonstrated the direct effects of ATOR on modulating the various steps of the TLR4 signaling pathway, other classic drug effects, such as cholesterol-lowering or alternative anti-inflammatory may have beneficial effects related to the development of cachexia syndrome. Having additional orthogonal approaches to modulate TLR4 signaling would be advantageous in validating our findings. However, statins have been reported to have some possible side effects including myopathy, which should be taken into account when considering their use in relation to the cachexia. Moreover, the exact mechanism of action of statins on *Tlr4* expression remains unknown. Although several papers show a downregulation in *Tlr4* using statins, further studies are needed to explore the exact molecular mechanisms underlying the regulation of statin-dependent *Tlr4* downregulation. Therefore, from a comprehensive and translational point of view, the effectiveness of pharmacologic inhibition of TLR4 by ATOR and other agents on the onset and progression of cancer cachexia, in particular the attenuation of the kinetics of tumor growth, still needs further study, especially in a clinical setting.

## Conclusion

**Figure 6:**
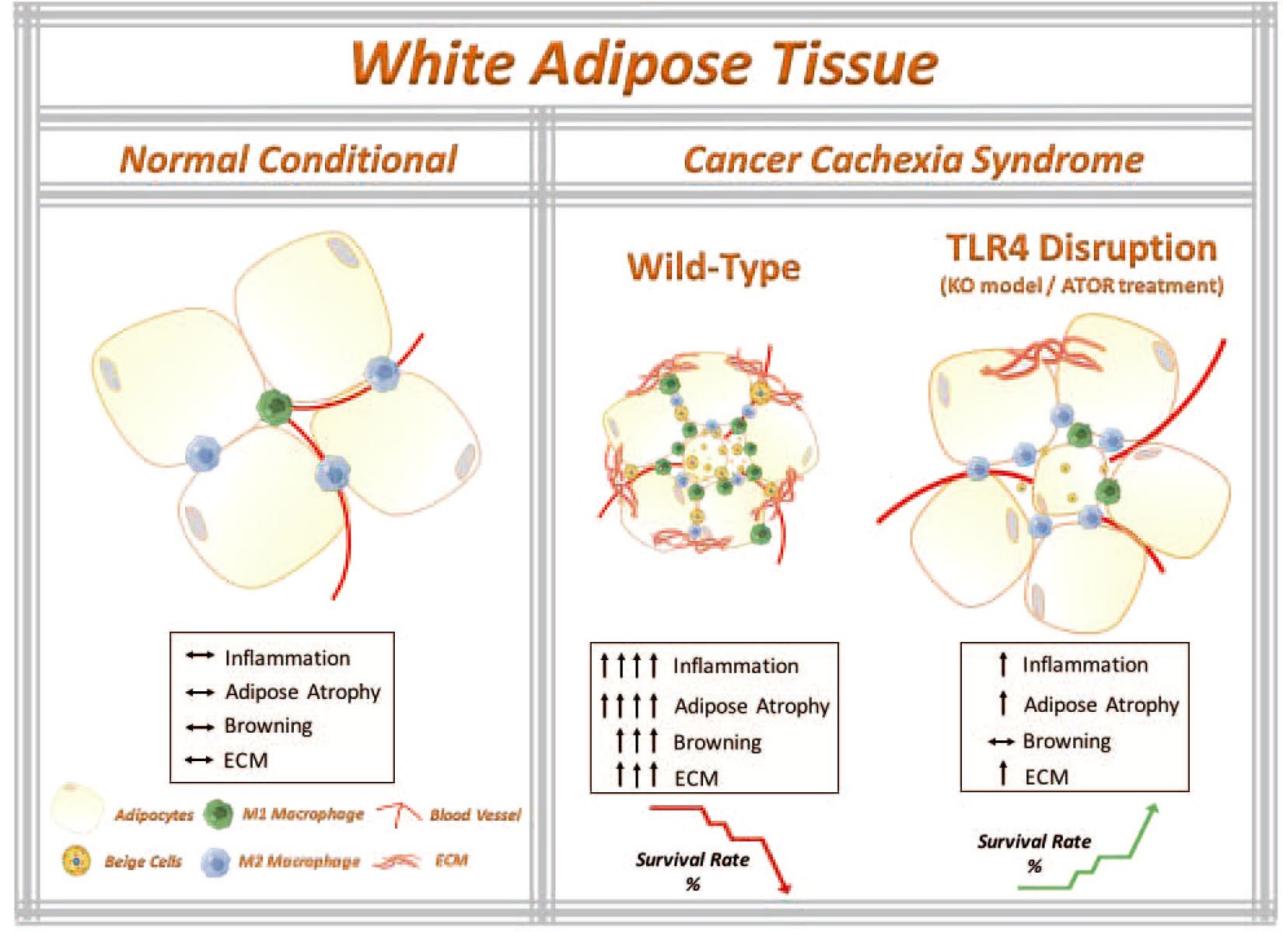
The working model that TLR4 disruption ameliorates adipose tissue remodeling during cancer cachexia syndrome. Cancer-induced cachexia is characterized by systemic inflammation, body weight loss and AT remodeling. We show that TLR4 disruption (knockout model of TLR4 and pharmacological inhibition by ATOR treatment) ameliorates AT remodeling, in particular, preservation of adipocyte atrophy and attenuation of browning phenotype in scAT, as well as inflammatory responses during cancer cachexia syndrome. Additionally, TLR4 disruption was effective in prolonging the survival and reducing tumor mass growth. Therefore, these data suggest that TLR4 plays an important role during cachexia development. Here, we suggested a new potential therapeutic target for cancer cachexia syndrome. Parts of the figure were drawn using images from Servier Medical Art. Servier Medical Art by Servier is licensed under a Creative Commons Attribution 3.0 Unported License (https://smart.servier.com/).

Our combined data from the TLR4 knockout model and ATOR treatment clearly shows that TLR4 plays an essential role in mediating tumor-induced AT remodeling and cachexia development. Therefore, disturbing TLR4 signaling systemically may prevent scAT remodeling by abrogating, at least in part, its indirect effects. At this point, both the genetic ablation and the pharmacological inhibition of TLR4 were able to attenuate both the remodeling of the AT (atrophy and inflammation) and the metabolic dysfunction (lipolysis) induced by cachexia (Fig. 6). Also, for the first time, we have presented evidence that the TLR4 receptor may play an essential role in the browning phenotype induced by cachexia. Interestingly, TLR4^−/−^ also showed resistance to browning, suggesting that the TLR4 receptor plays an essential role in the morphological alterations and homeostatic energy expenditure. Finally, it is clinically noteworthy that animals treated with a TLR4 inhibitor displayed general attenuation of AT remodeling and systemic inflammation (Fig. 6), presenting an increase in the mice survival, which may have a relevant role as an adjuvant treatment option, as well as being a strong candidate for novel anti-cachectic therapy development.

## Methods

### Animal Model

Eight-week-old male C57BL/6 J and TLR4^−/−^ (Toll-like Receptor 4 - B6.B10ScN-Tlr4lps-del/JthJ - JAX stock #007227) mice were obtained from Jackson Laboratory, for validation and phenotypic characterization of TLR4^−/−^ see Fig. S4. Mice were housed on a 12 h light/dark schedule and had free access to water and food. Mice were divided into four different groups: Wild Type (WT); WT tumor-bearing (WT TB); TLR4^−/−^ and TLR4^−/−^ tumor-bearing (TLR4^−/−^ TB). For chronic cold challenge, WT and TLR4^−/−^ mice were single-caged and housed at room temperature (23 °C) and cold exposure (6 °C) for six days (n = 5 per genotype and per condition) on a 12 h light/12 h dark cycle with access to a standard chow diet. All of the studies performed were approved by the Ethical Committee for Animal Research from the University of Mogi das Cruzes approved all the adopted procedures, which were carried out in accordance with the ethical principles stated by the Brazilian College of Animal Experimentation - Protocol n. 009/2013.

### Tumor Model and Collection of Tissues

Lewis Lung Carcinoma (LLC) tumor cells were used for inducing cancer cachexia syndrome. LLC was injected subcutaneously into the right flanks (200 μl LLC cells −3.5 × 10^5^). Non-tumor bearing control mice received Phosphate-buffered saline (PBS) only. Development of cachexia was monitored by tumor size and body weight for 28 days. The impact of tumor growth and development of cachexia on animal welfare were considered, with appropriate measures to monitor and alleviate suffering implementation according to adopted endpoint criteria^9,48^. Overnight-fasted mice were euthanized by decapitation without anesthesia, serum was collect and centrifuged (400 g, 15 min, 4 °C) and stored at −80 °C for later analysis. After euthanasia, subcutaneous adipose tissue (scAT) was carefully dissected and weighed. All the mice were checked if they are positive for the presence of metastasis in the lungs, as well as other visceral organs. If there is a presence of metastasis, the animals are excluded from the experimental group (exclusion criteria). Body weight was measured daily, at the same time, over the 28-day experimental period on a precision scale (Ohaus®).

### Atorvastatin Treatment

For the inhibition of the TLR4 receptor via pharmacological action, Atorvastatin (Citalacor®) treatment was performed, currently described as a selective antagonist for TLR4. Only WT TB mice were used for this procedure. The animals were divided into two experimental groups: WT TB + PBS and WT TB + Atorvastatin (TB + ATOR). The animals that received Atorvastatin were treated with a concentration of 10 mg/kg/day by orogastric gavage during the 27-day of protocol. The same protocol was performed for the animals that underwent the survival experiment.

### Histological analysis

scAT was fixed in HistoChoice® MB (Amresco), pH 7.4 for 3 h. Fixed tissues were dehydrated and 8-μm sections were used. H&E and Picrosirius staining were performed according to standard procedures (Sigma-Aldrich). The sections were analyzed with a Leica microscope (DM 750). Morphometric aspects were measured by Imagen Pro-Plus 6.0.

### Immunohistochemistry

Immunohistochemistry of scAT was carried out with sections fixed by HistoChoice® MB (Amresco) at pH 7.4 for 3 hours. Fixed tissues were dehydrated and 5-μm sections were used. Dehydrated tissue was blocked using a two different blocking buffers, a buffer solution of endogenous peroxidase activity with 0.3% H_2_O_2_ in methanol and buffer solution of free protein binding sites with 5% normal goat serum. The following primary antibodies were used: UCP1 (ab10983), TNFα (ab6671), CD68 (ab955) and CD3 (ab16669). We employed the polymer-peroxidase method, using Histofine Simple Stain MAX-PO (Nichirei Biosciences) and Sigma Fast 3,3-diaminobenzidine as substrate (Sigma-Aldrich). Sections were counter-stained with hematoxylin.

### RNA isolation and qRT-PCR

Total RNA was isolated from scAT using QIAzol Lysis Reagent Protocol (QIAGEN) following the manufacturer’s instructions. cDNA was synthesized from 2 μg of total RNA using iScript cDNA Synthesis Kit (BioRad). RPL19 served as control for internal reference gene. Primer sequences used for qRT-PCR analyses were listed in Table S2. Analyses of qRT-PCR products were performed with the Prism 7500 SDS software (Thermo Fisher Scientific). Relative quantification of mRNA amount was obtained by the by 2^−(ΔΔCt)^ method.

### Western Blot

For protein expression analyses, tissues were homogenized in lysis buffer (20 mM HEPES, 150 mM NaCl, 2 mM EDTA, 1% Triton X-100, 0.1% SDS, 10% glycerol, 0.5% sodium deoxycholate) supplemented with Halt protease and phosphatase inhibitors (Thermo Pierce). Samples from tissue lysates were then resolved by SDS-PAGE, and immunoblots were performed using standard protocols. Membranes were blotted with the following antibodies: HSL (ab45422); phospho-HSL Ser660 (Cell Sig. #4126); ATGL (Cell Sig. #2138); P38 MAPK (Cell Sig. #8690); phospho P38 MAPK (Cell Sig. #9211); phospho-AMPK (Thr172 - Cell Sig. 2531); AMPK (Cell Sig. 2532); phospho-PKA substrate (Cell Sig. #9624); Adiponectin (ab62551) and UCP1 (ab10983), all overnight. The membranes were incubated with the goat anti-rabbit IgG conjugated to horseradish peroxidase secondary antibody (7074 - Cell Signaling Technology) for 1 h at room temperature and were detected by ECL Prime (Amersham). For the control of protein loading and transfer, we used Ponceau staining of the membranes. Quantification of the antigen–antibody complex was performed by the ImageJ Analysis Software.

### SVF isolation and Flow cytometry analysis

Stroma vascular fraction (SVF) from scAT were excised and minced in PBS with calcium chloride and 0.5% BSA. Collagenase II (Sigma-Aldrich) was added to 1 mg/ml and incubated at 37 °C for 30 minutes with shaking. The cell suspension was filtered through a 100 μm filter and then spin down at 300 g for 5 minutes to separate floating adipocytes from the SVF pellet. Cells were incubated with Fc Block (BD Biosciences) prior to staining with conjugated antibodies for 15 minutes at 4 °C. SVF were purified and stained with the following antibody panel: ATMϕs were defined as CD68^+^F4/80^+^ subpopulations. M1 and M2 ATMϕs were defined as CD68^+^F4/80^+^CD11c^+^CD206^−^ and CD68^+^F4/80^+^CD11c^−^CD206^+^, respectively. Cells were analyzed using a BD Accuri (BD biosciences). The data was analysis was performed using FlowJo.

### Measurement of plasma cytokines

Plasma was collected using plasma separators (BD Biosciences). Cytokine levels were determined using a Milliplex assay kit (Milliplex MAP mouse cytokine/chemokine magnetic bead panel, #MCYTOMAG-70K - Millipore). Data were analyzed using the Bio-Plex manager software (Bio-Rad Laboratories).

### Lipolysis assay

Isolated adipocytes were suspended in 40 ml of Kreb’s/Ringer/phosphate buffer (pH 7.4, with 1% BSA) in 0.6 ml microtubes with 20 ml of adenosine deaminase (0.2 U/ml) and were incubated at 37 °C for 5 min to degrade endogenously released adenosine, which is a potent inhibitor of lipolysis. The cells were then incubated for 1 h at 37 °C either in the presence (stimulated) or absence (basal) of isoproterenol (10^−5^M, β-adrenergic agonist) in a final volume of 200 μL. At the end of incubation, the reaction mixture was blocked by moving the tubes to a cold-water bath followed by centrifugation at 3500 g for 5 min at 4 °C. The infranatant was carefully transferred to microtubes containing 150 μL of silicone oil and re-centrifuged at 3500 g for 2 min. The concentration in the medium of released glycerol (nmol/10^4^ cells) was quantified with a commercially available reagent (Sigma-Aldrich), measured according to the manufacturer’s instructions. The lipolysis response was assessed by the ratio of glycerol released relative to the basal condition. For additional analyses of the possible effect of ATOR on inhibiting lipolysis, 3T3-L1 cells were stimulated during 1 h, 6 h and 24 h with lipopolysaccharide (LPS, 100 ng/mL). The concentration in the medium of released glycerol was quantified with a commercially available reagent (Sigma-Aldrich), measured according to the manufacturer’s instructions.

### Cell culture

3T3-L1 pre-adipocytes were plated at 1 × 10^4^ in 24-well culture plates and cultured in DMEM (Dulbecco’s Modified Eagle’s Medium) with 4500 mg glucose supplemented with 10% bovine serum and 2% penicillin with streptomycin at pH 7.4. The cells were maintained at 37 °C with 5% carbon dioxide (CO_2_) so as not to reach complete confluence until they were induced to differentiate. Preadipocytes were brought to complete confluence (day-2) and after two days of confluence, the culture medium was replaced by differentiation inducer medium, consisting of DMEM, supplemented with 10% fetal bovine serum (FBS), 1 μM dexamethasone, 0.5 mM 3-isobutyl-1-methylxanthine (IBMX) and 1.67 μM bovine insulin. From the second day of differentiation, the cells were maintained in culture medium containing only 0.83 μM insulin and 10% FBS which was changed every 48 hours for eight days.

### Adipose Tissue lipid analysis

The total scAT lipids were extracted using the method of Folch^49^. Transesterification of the lipid extracts was performed with acetyl chloride (5% CH_3_COCl in methanol) and fatty acids (FAs) composition was determined as methyl esters using a Varian Model 3900 Gas Chromatograph (GC) coupled to flame ionization detection (FID). FAs were identified by comparing the retention time using three known standards of fatty acid methyl esters (FAME). The FAMEs were analyzed in a capillary column (CP Wax 52 CB), 0.25 mm in thickness, 0.25-μm inside diameter, and 30 m in length. Hydrogen was used as a carrier gas at a linear velocity of 22 cm/s. The temperature program was 170 °C for 1 min followed by a 2.5 °C/min ramp to 240 °C, and a final hold time of 5 min. Injector and FID temperatures were 250 and 260 °C, respectively. The profiles of individual FAs were calculated using an automatic integrator and presented as percentages of total FAs according to the peak areas. Data are presented as palmitic acid profile (%) of total lipids.

### Non-Esterified Fatty Acids (NEFA)

50 μL of serum was collected from the different groups and the levels of NEFA was quantified using a kit following the manufacturer’s recommendations (MAK044, Sigma).

### Statistical analysis

Data were analyzed in GraphPad Prism 7 (GraphPad Software). The statistical significance of the differences in the means of experimental groups was determined by Student’s t-test or and 2-way ANOVA, followed by Tukey’s post hoc comparison tests. The data are presented as means ± SEM. P values ≤ 0.05 were considered significant.

## Electronic supplementary material

Supplementary Information
