# Supplementary material for: Toll-Like Receptor-4 Disruption Suppresses Adipose Tissue Remodeling and Increases Survival in Cancer Cachexia Syndrome
Source: Sci Rep. 2018 Dec 21;8:18024. doi: 10.1038/s41598-018-36626-3 (PMC6303407; doi:10.1038/s41598-018-36626-3)

Toll-Like Receptor-4 Disruption Suppresses Adipose Tissue Remodeling and Increases Survival in Cancer Cachexia Syndrome

Felipe Henriques[1,2], Magno A. Lopes[1], Felipe O. Franco[1], Pamela Knobl[1], Kaltinaitis B. Santos[1], Luana L. Bueno[1], Victor A. Correa[1], Alexander H. Bedard[2], Adilson Guilherme[2], Alexander Birbrair[3], Sidney B. Peres[4], Stephen R. Farmer[5] and Miguel L. Batista Jr[1, *].

[1] Integrated Group of Biotechnology, Laboratory of Adipose Tissue Biology, University of Mogi das Cruzes, São Paulo, Brazil;

[2] Program in Molecular Medicine, University of Massachusetts Medical School, Worcester, Massachusetts, USA;

[3]Department of Pathology, Federal University of Minas Gerais, Minas Gerais, Brazil;

[4]Department of Physiological Sciences, State University of Maringá, Paraná, Brazil;

[5]Department of Biochemistry, Boston University School of Medicine, Boston, Massachusetts, USA;

[*]**Corresponding author**: Laboratory of Adipose Tissue Biology, University of Mogi das Cruzes, Av. Dr. Cândido Xavier de Almeida Souza, 200, Vila Partênio, Mogi das Cruzes, SP, 08780-911, Brazil. Tel.: +55 11 3091 7225; Fax: +55 11 3091 7402.

E-mail: migueljr4@me.com

Supplementary Table 1. General cachexia Parameters: Tumor Bearing (TB) mice and TB+ATOR treatment during the experimental protocol.

Morphological Parameters	TB	TB+ATOR	P
Carcass (g)	22.02 ± 0.46	23.16 ± 0.76	0.203
BW increase (%)	-0.23 ± 0.38	-0.01 ± 0.40	0.014
BW plateau (Days)	21.54 ± 0.81	28.49 ± 1.59	0.006
Cachexia Index	14.18 ± 1.97	10.95 ± 1.49	0.145
Tumor Appearance (Days)	13.56 ± 0.32	16.56 ± 0.87	0.003

TB, Tumor Bearing; TB+ATOR, Tumor Bearing+ATOR treamtent; BW, body weight. Graphs show the mean ± SEM. Statistical significance was determined by Student's t-test (TB vs.TB+ATOR).

Supplementary Table 2. Primer sequences used in RT-qPCR analysis.

GENE	Forward	Reverse
UCP-1	GGG ACG TCA TCT GCC AGT	GAA AGG GAC GAC CCC TAA TC
PGC-1α	GGA GGA GTT GTG GGA GGA GT	AGT CAC CAA ATG ACC CCA AG
PRDM16	ACC ATG GGC TTT GAC CAT AC	GAC GAG GGT CCT GTG ATG TT
CIDEA	TCT GCA ATC CCA TGA ATG TC	TAA GAG ACG CGG CTT TGG
CIDEC	CAC TGC TAC AAG GCC AAG C	ATG TGA CTG GAG GTG CCA AG
ADRB1	GCA TCA TCA TGG GTG TGT TC	GAA GAC GAA GAG GCG ATC C
ADRB3	CAA GCT TCC TTG CTG GAT CT	CTG TTG AAG CCA GGC AGA GT
GLUT4	GTG ACT GGA ACA CTG GTC CTA	CCA GCC ACG TTG CAT TGT AG
PCK1	CTG CAT AAC GGT CTG GAC TTC	CAG CAA CTG CCC GTA CTC C
ACSL1	TGC CAG AGC TGA TTG ACA TTC	GGC ATA CCA GAA GGT GGT GAG
ACADS	CTA CTG TGC TTC AGG GAC AAC	CAA AGG ACT TCG ATT CTG CCC
CS	GGA CAA TTT TCC AAC CAA TCT GC	TCG GTT CAT TCC CTC TGC ATA
ME3	GGA ACC CCC ATC TCA ACA AGG	GTT GCG GTC TTG GTG AGT CA
CPT1B	GCA CAC CAG GCA GTA GCT TT	CAG GAG TTG ATT CCA GAC AGG TA
LPL	GGG AGT TTG GCT CCA GAG TTT	TGT GTC TTC AGG GGT CCT TAG
PERILIPIN	GGG ACC TGT GAG TGC TTC C	GTA TTG AAG AGC CGG GAT CTT TT
NOS2	GTT CTC AGC CCA ACA ATA CAA GA	GTG GAC GGG TCG ATG TCA C
CD11C	CTG GAT AGC CTT TCT TCT GCT G	GCA CAC TGT GTC CGA ACT CA
CD301	TGA GAA AGG CTT TAA GAA CTG GG	GAC CAC CTG TAG TGA TGT GGG
ARG1	CTC CAA GCC AAA GTC CTT AGA G	AGG AGC TGT CAT TAG GGA CAT C
RPL19	GGG AAG AGG AAG GGT ACT GC	GAC GGT CAA TCT TCT TAG ATT CC
$TLR4^{-/-}$	GCA AGT TTC TAT ATG CAT TCT C	CCT CCA TTT CCA ATA GGT AG
WT	ATA TGC ATG ATC AAC ACC ACA G	TTT CCA TTG CTG CCC TAT AG

Supplementary Figure 1. ***Evaluation of tumor mass in both tumor groups.*** Wild-type C57BL/6 and TLR4$^{-/-}$ mice (8-week-old male) were inoculated with LLC cells. Tumor mass in WT and TLR4$^{-/-}$ mice after experimental protocol (27 days after cells inoculation). N = 10 per group. Graphs show the mean ± SEM. ns = not significant.

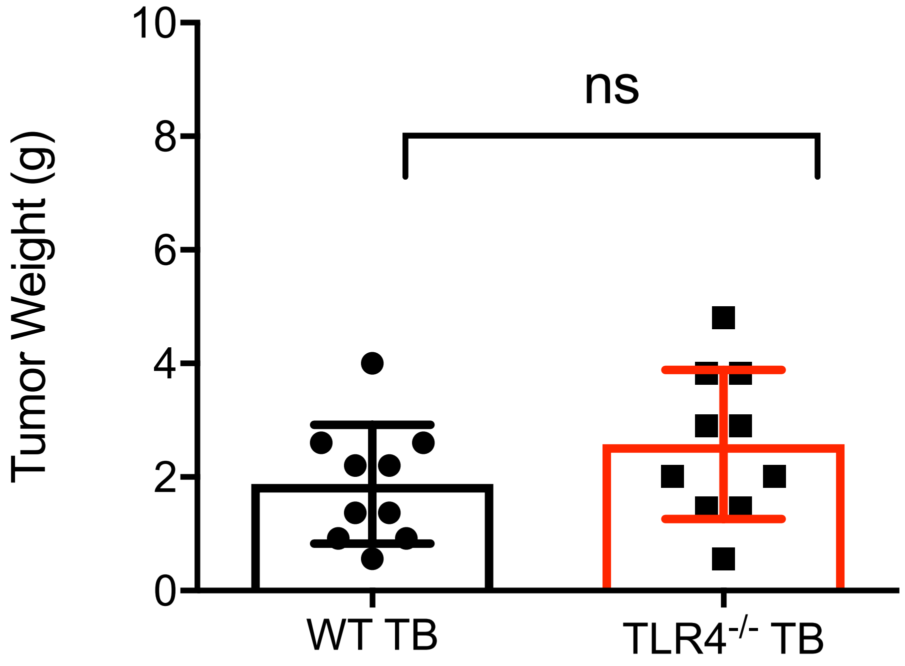

Supplementary Figure 2. ***No difference in phospho-PKA substrates.*** Representative immunoblots for phospho-PKA substrate (Cell Sig. #9624) in different experimental conditions.

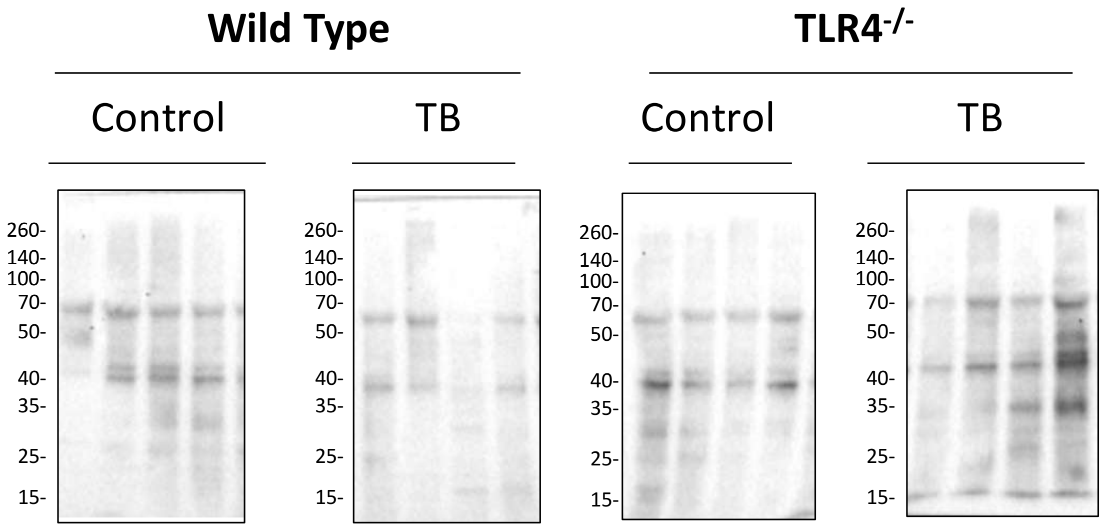

Supplementary Figure 3. ***Atorvastatin treatment inhibit Tlr4 mRNA expression.*** The animals that received Atorvastatin were treated with a concentration of 10 mg/kg/day by orogastric gavage during the 27-day of protocol. The same protocol was performed for the animals that underwent the survival experiment. qRT-PCR was performed to *Tlr4 and Nfkb* for mRNA quantification in scAT from TB and TB+ATOR. N = 5 per group. Graphs show the mean ± SEM. Statistical significance was determined by Student's *t*-test. ***P < 0.001.

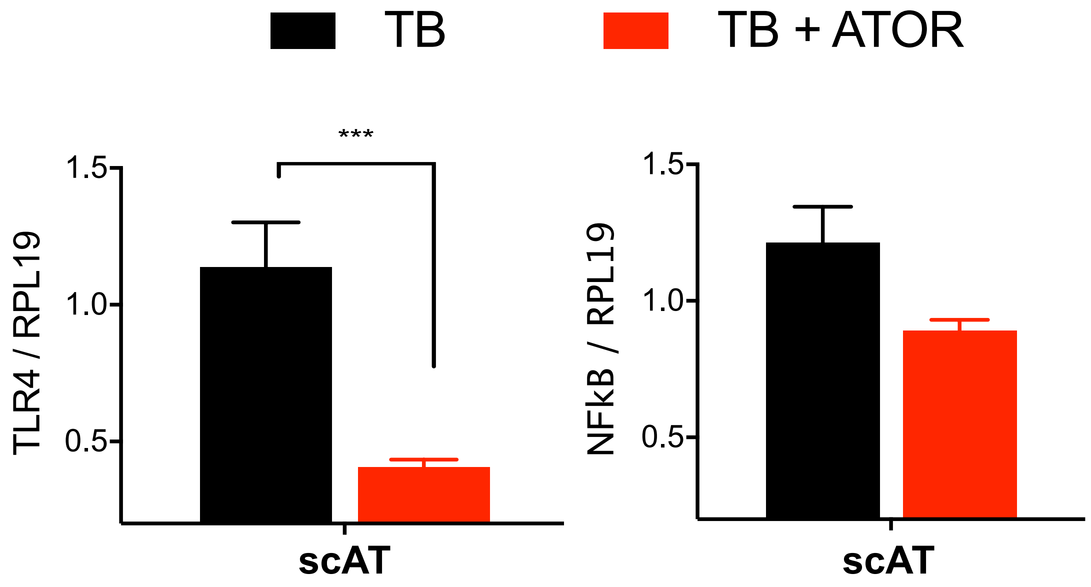

Supplementary Figure 4. ***Validation that TLR4 is absent in the TLR4 knockout mice.*** Representative image of the genotype identification. **(A)** Genotyping was initially conducted by PCR on DNA extracted from the heterozygous mice. An Agarose gel show PCR products with 390pb (WT), 390pb and 140pb (heterozygous) and 140pb TLR4$^{-/-}$. Lane 1 and 2, Wild-Type (+/+); Lane 3-8, Heterozygous (+/-); Lane 9, TLR4$^{-/-}$ (-/-). **(B)** Further confirmation of the genotype for TLR4$^{-/-}$ mice was obtained by qRT-PCR. *Tlr4* mRNA levels was quantified in scAT from WT and TLR4$^{-/-}$ mice. N = 5 per group. Graphs show the mean ± SEM. Statistical significance was determined by Student's *t*-test. ***P < 0.001.

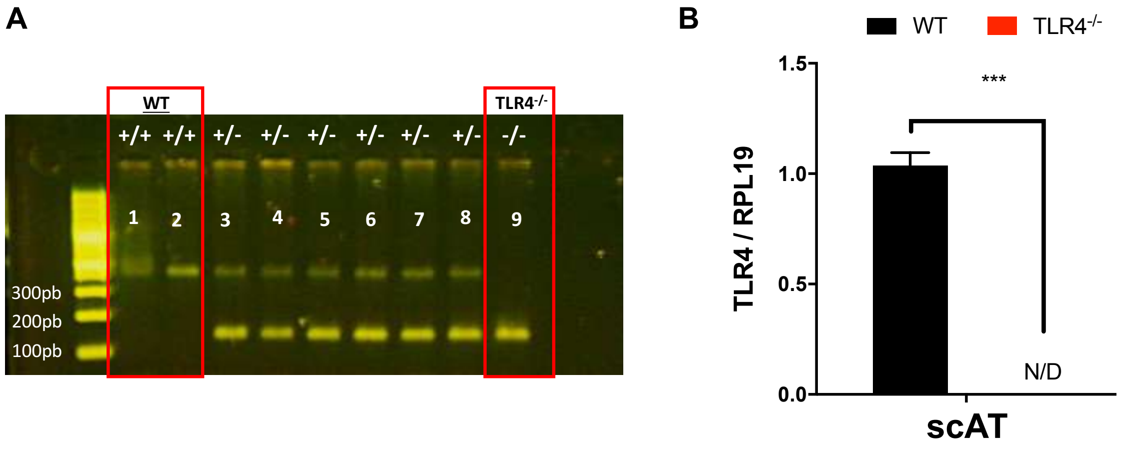

Supplementary Figure 5. Original membranes for the representative Western Blots shown in Figure 2, Figure 3, Figure 4 and Supplementary Figure 2.

Phospho HSL-Ser660

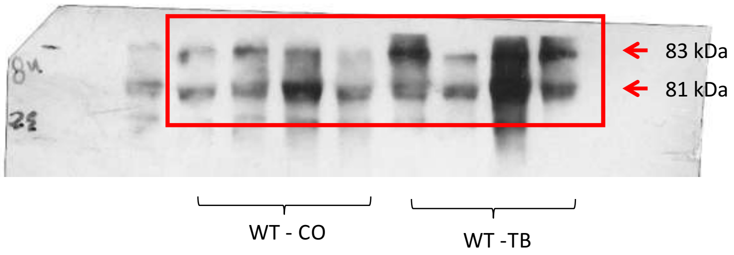

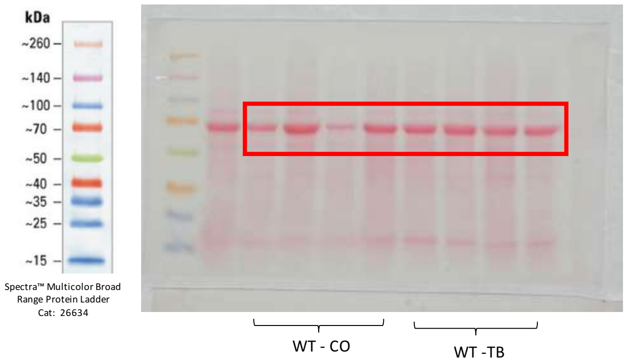

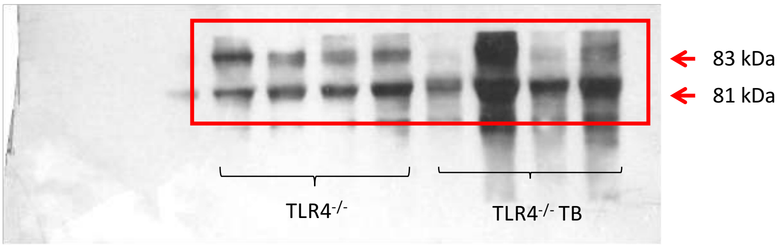

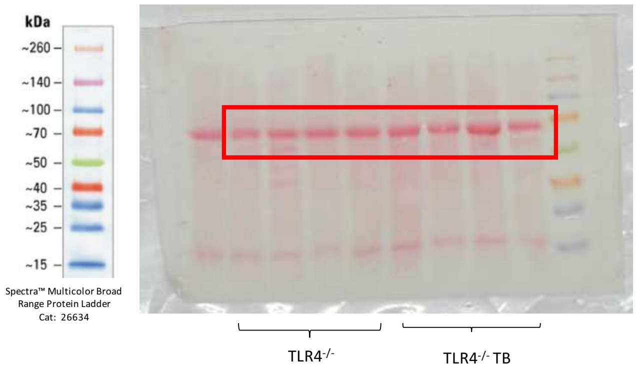

Total HSL

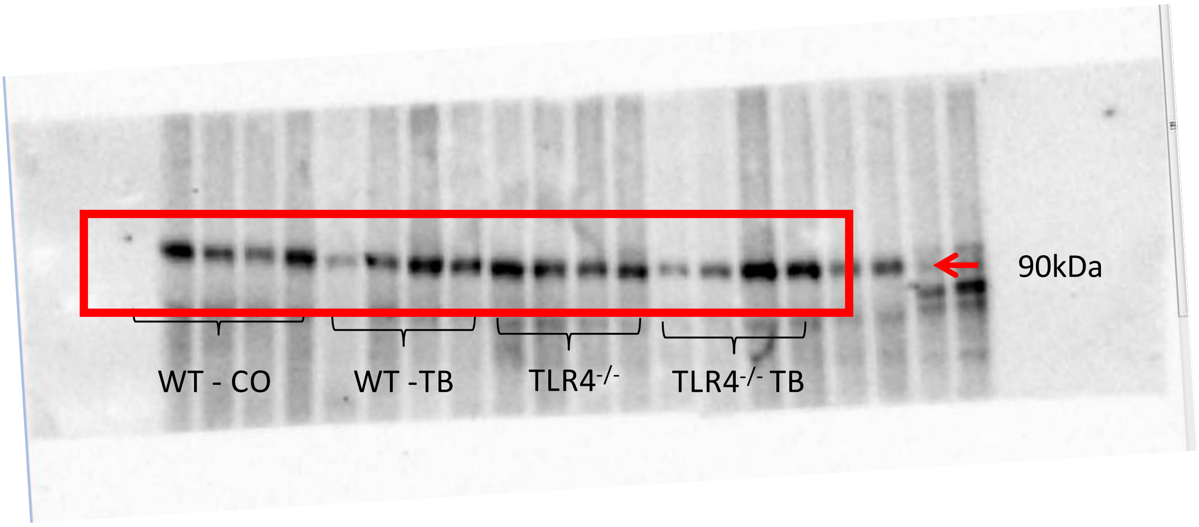
90kDa
WT - CO
WT -TB
TLR4-/-
TLR4-/- TB

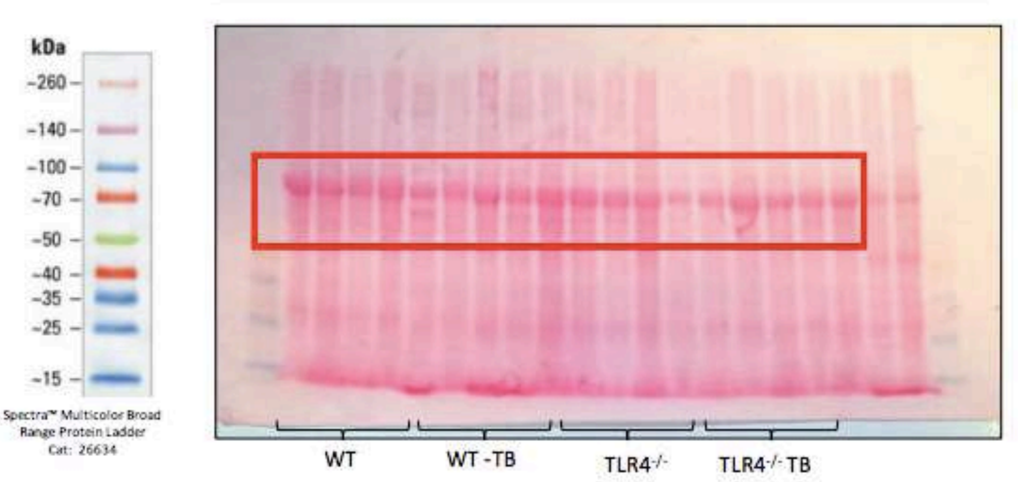
kDa
-260
-140
-100
-70
-50
-40
-35
-25
-15
Spectra™ Multicolor Broad Range Protein Ladder
Cat: 26634
WT
WT -TB
TLR4-/-
TLR4-/- TB

Phospho P38 MAPK

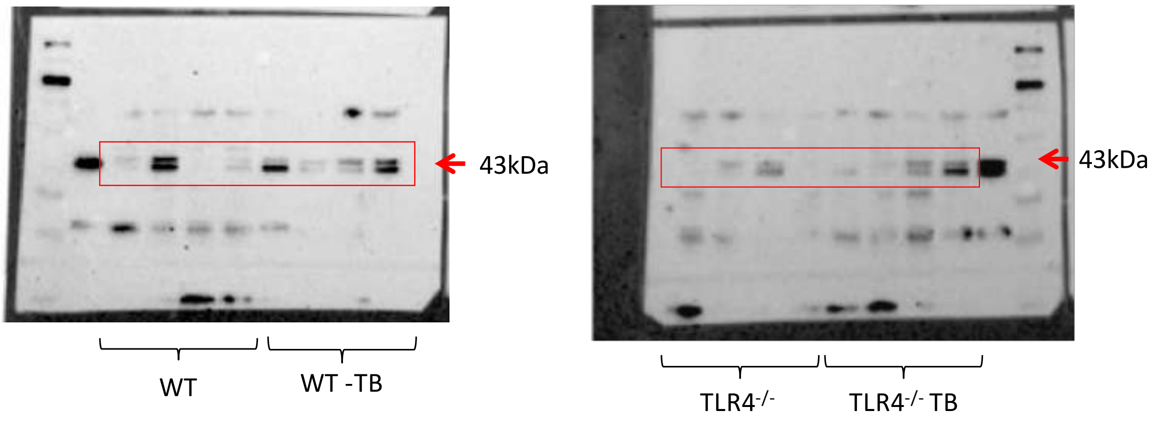

Total P38 MAPK

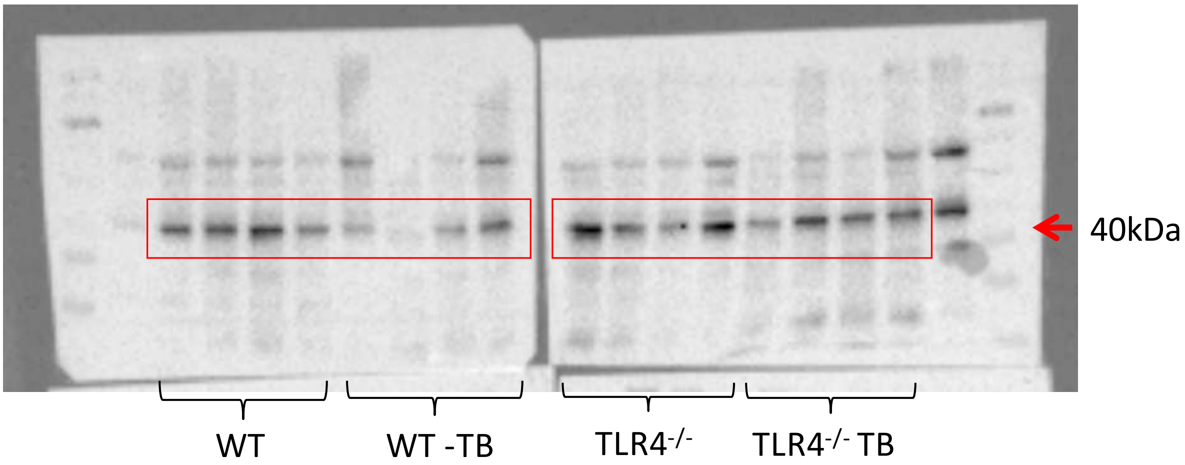

Phospho AMPK

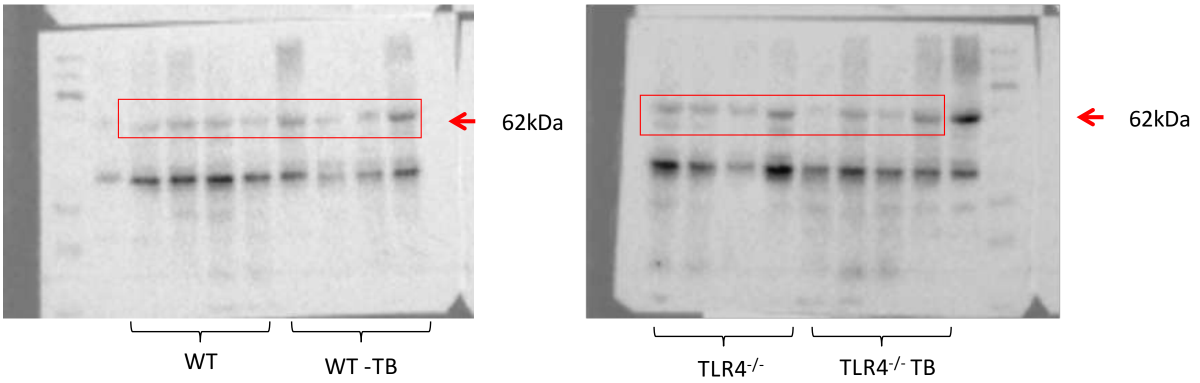

Total AMPK (Short Exposure)

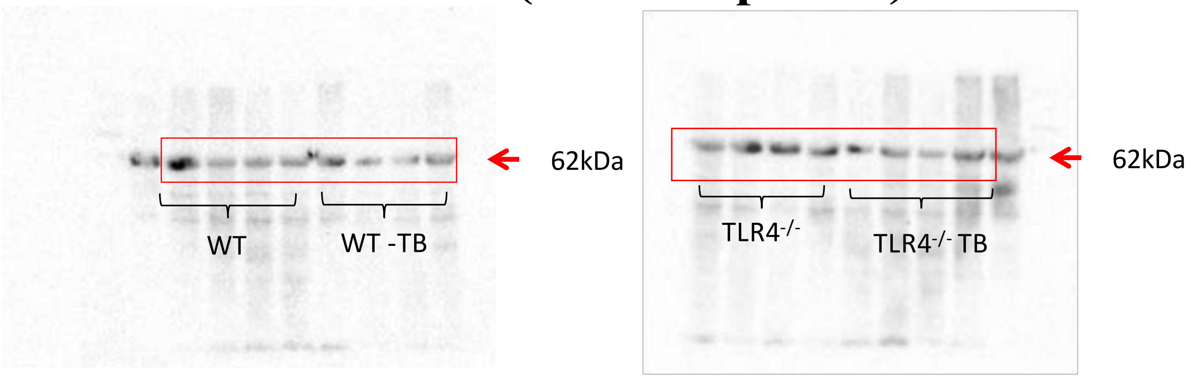

Total AMPK (Long Exposure)

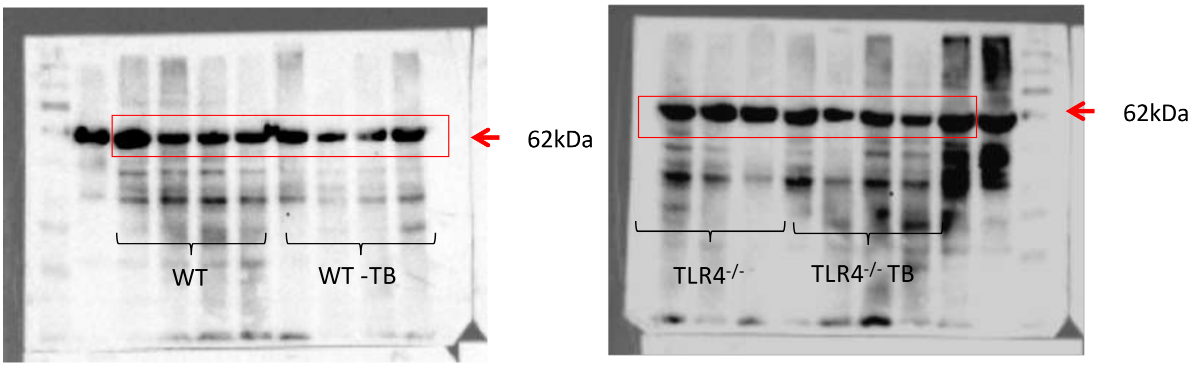

Phospho HSL-Ser660

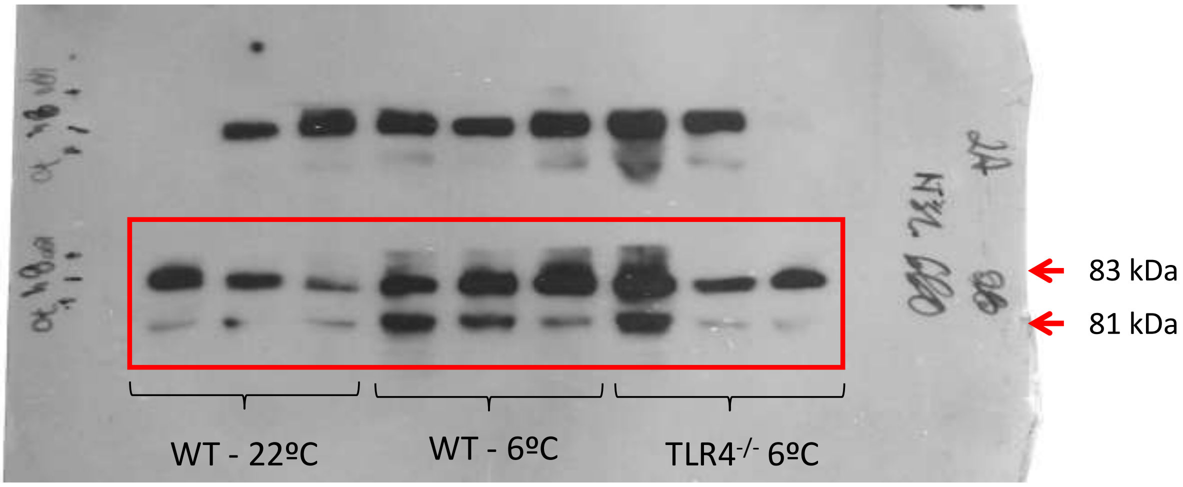

Total HSL

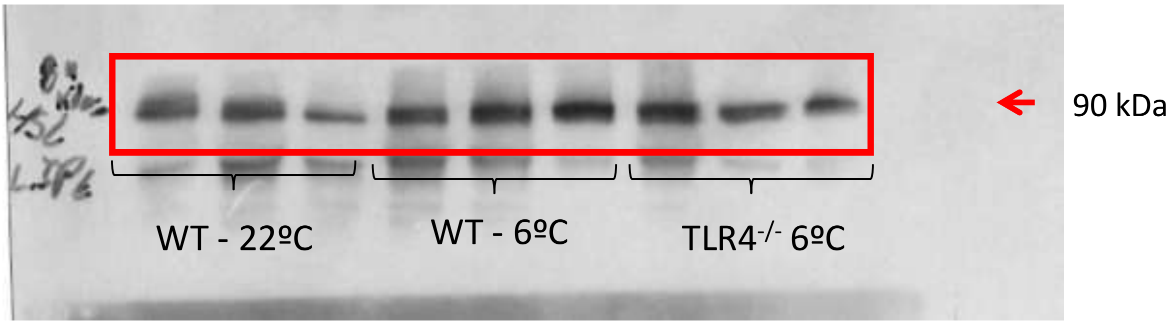

UCP1

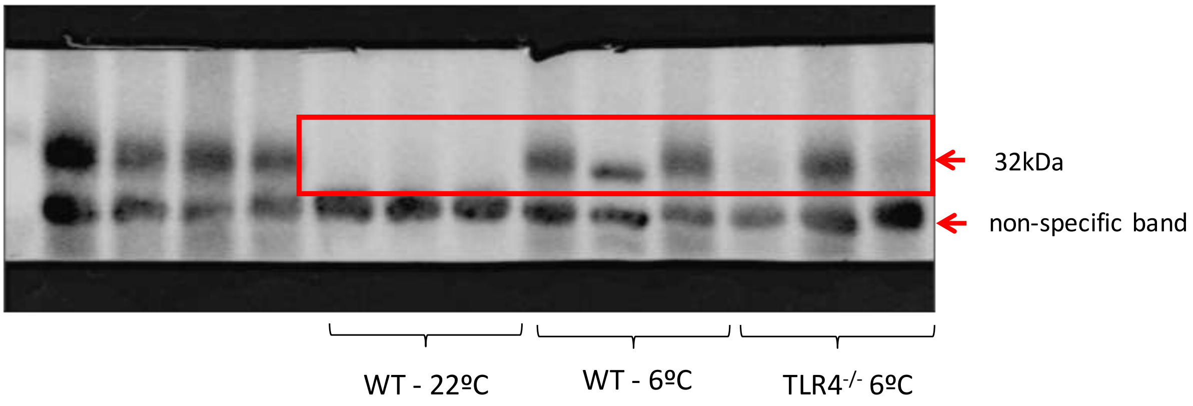
32kDa
non-specific band
WT - 22ºC
WT - 6ºC
TLR4-/- 6ºC

ATGL

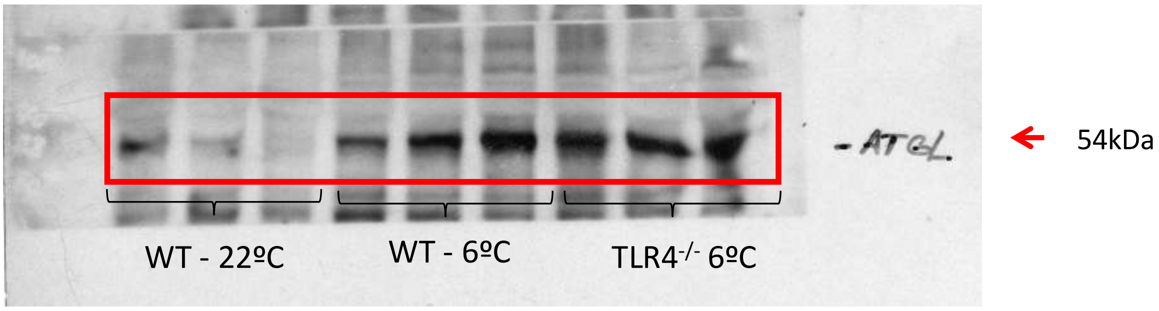
ATGL
54kDa
WT - 22ºC
WT - 6ºC
TLR4-/- 6ºC

Adiponectin

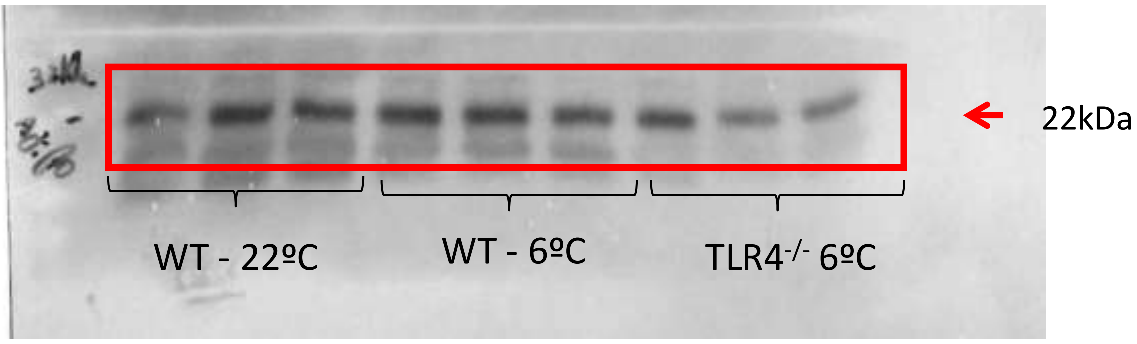

Ponceau

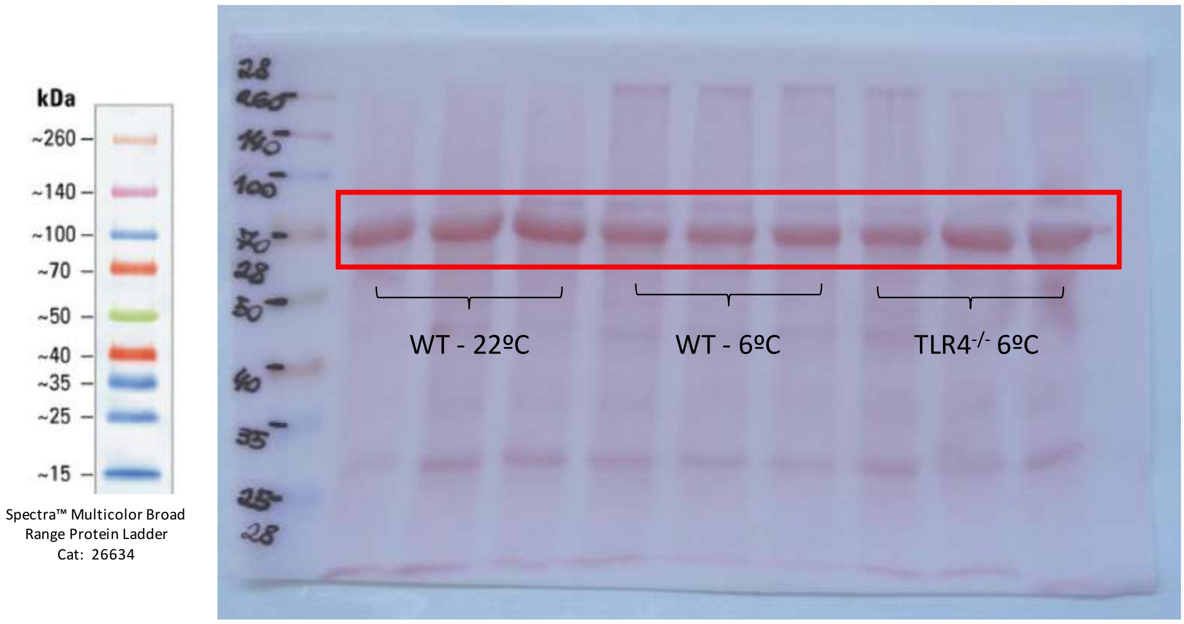

Phospho PKA Substrates

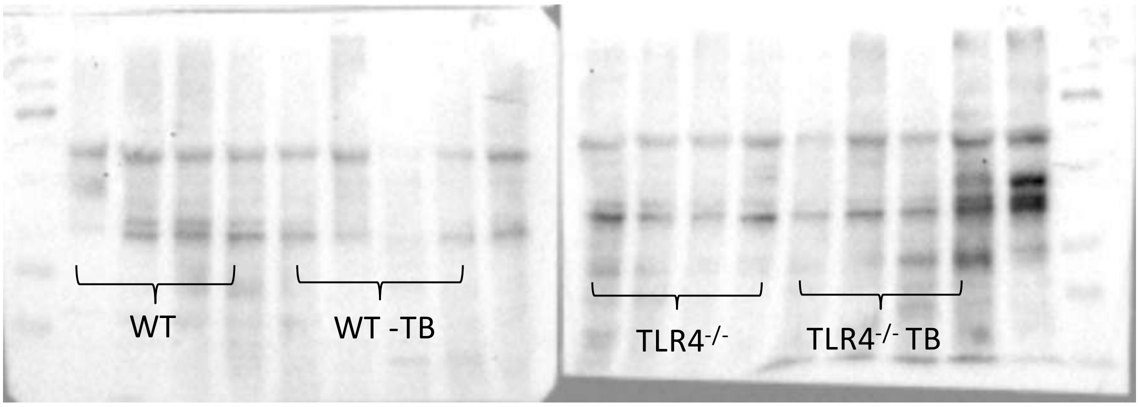

Supplement: Supplementary file 1 — Supplementary Information [file 41598_2018_36626_MOESM1_ESM.pdf]
